# Atomic Intercalation Induced Spin-Flip Transition in Bilayer CrI_3_

**DOI:** 10.3390/nano12091420

**Published:** 2022-04-21

**Authors:** Dongsi Wu, Ying Zhao, Yibin Yang, Le Huang, Ye Xiao, Shanshan Chen, Yu Zhao

**Affiliations:** 1 School of Materials and Energy, Guangdong University of Technology, Guangzhou 510006, China; 2112002016@mail2.gdut.edu.cn (D.W.); yingzhao021012@163.com (Y.Z.); yangyibin@gdut.edu.cn (Y.Y.); huangle@gdut.edu.cn (L.H.); yexiao@gdut.edu.cn (Y.X.); zhaoyu@gdut.edu.cn (Y.Z.); 2 Guangdong Provincial Key Laboratory of Information Photonics Technology, Guangdong University of Technology, Guangzhou 510006, China

**Keywords:** density functional theory, atomic intercalation, spin-polarization, superexchange

## Abstract

The recent discovery of 2D magnets has induced various intriguing phenomena due to the modulated spin polarization by other degrees of freedoms such as phonons, interlayer stacking, and doping. The mechanism of the modulated spin-polarization, however, is not clear. In this work, we demonstrate theoretically and computationally that interlayer magnetic coupling of the CrI_3_ bilayer can be well controlled by intercalation and carrier doping. Interlayer atomic intercalation and carrier doping have been proven to induce an antiferromagnetic (AFM) to ferromagnetic (FM) phase transition in the spin-polarization of the CrI_3_ bilayer. Our results revealed that the AFM to FM transition induced by atom intercalation was a result of enhanced superexchange interaction between Cr atoms of neighboring layers. FM coupling induced by O intercalation mainly originates from the improved superexchange interaction mediated by Cr 3*d*-O 2*p* coupling. FM coupling induced by Li intercalation was found to be much stronger than that by O intercalation, which was attributed to the much stronger superexchange by electron doping than by hole doping. This comprehensive spin exchange mechanism was further confirmed by our results of the carrier doping effect on the interlayer magnetic coupling. Our work provides a deep understanding of the underlying spin exchange mechanism in 2D magnetic materials.

## 1. Introduction

The discovery of magnetic ordering in two-dimensional (2D) semiconductors has led to increased interest in both fundamental physics and potential applications [1,2,3,4]. Among these 2D magnetic materials, CrI_3_ is the most intensively studied due to its intrinsic intralayer ferromagnetic (FM) coupling and interlayer antiferromagnetic (AFM) coupling [5]. The rather weak magnetic coupling in most 2D magnets has become a significant obstacle for applications in spintronic devices. Hence, substantial research efforts have been made to enhance the magnetic coupling in 2D magnetic materials. Doping [6], defect engineering [7], and dimensionality reduction [8] are promising strategies to enhance the magnetic coupling by introducing highly localized orbitals. Some other 2D materials with intrinsic FM ordering such as Cr_2_Ge_2_Te_6_ [9] and CrBr_3_ [10] have been developed experimentally or predicted theoretically. This research has provided exciting new opportunities for applications of 2D magnetic materials in spintronics.

As one of the first discovered 2D FM materials, CrI_3_ has attracted much attention [5]. The ferromagnetism of single-layer CrI_3_ has been widely studied. However, its Curie temperature T_C_ is much lower than room temperature [5]. A pivotal issue is tailoring its magnetism through practical methods such as strains [11,12,13,14], carrier doping [15,16], defect engineering [17,18,19,20,21], applying external fields [22,23,24], and surface adsorption [25,26,27,28,29,30]. Specifically, Zheng et al. applied strains to single-layer CrI_3_ and found a phase transition from FM to antiferromagnetic (AFM). They also found that tensile strain can tune its easy magnetization axis to the in-plane direction from the original out-of-plane direction [31]. Lithium (Li), as a typical donor defect, has been widely used to engineer the electronic properties of 2D materials [32,33,34,35]. It also has been proven that the adsorption of Li atoms can further enhance FM spin-polarization of monolayer CrI_3_ and further increase the Curie temperature [29]. However, the mechanism of manipulating magnetism in 2D CrI_3_ is has not been well revealed.

Theoretical understanding of tunable magnetism in 2D magnets is of great importance for their device applications. Previous work revealed that the intralayer FM and interlayer AFM order in the CrI_3_ bilayer are governed by direct-exchange and superexchange interactions, respectively [36]. A further work demonstrated that the interlayer FM order was favored by the *e_g_*–*t_2g_* interactions and AFM order was favored by the *e_g_–e_g_* and *t_2g_–t_2g_* interactions [37]. X. Chen et al. also attributed the magnetic coupling in 2D CrSiTe_3_ to a combined effect of AFM direct-exchange interaction and FM superexchange interaction [38] while it is generally accepted that direct-exchange interaction usually occurs in metallic magnets. It can be concluded from previous works that the *e_g_–e_g_* and *t_2g_–t_2g_* exchange interactions are responsible for the AFM state and the *e_g_–t_2g_* hopping channels result in the FM state. While a general model is lacking, the type of intralayer magnetic coupling in the CrI_3_ bilayer is still under debate. Furthermore, these works also proved that interlayer magnetic coupling in these 2D magnets showed sensitive modulation by strains, carrier doping, and stacking form. How the magnetic coupling is tuned by these strategies is not well understood yet.

In this context, we performed first-principles calculations on layered CrI_3_ to explore the intralayer and interlayer magnetic coupling. It is proposed that the intralayer FM coupling in CrI_3_ mainly originates from the *e_g_*–*t_2g_* superexchange interactions. The competition between *e_g_–e_g_*, *t_2g_–t_2g_* interactions, and *e_g_*–*t_2g_* interactions gives rise to the stacking-tunable interlayer magnetic order in the CrI_3_ bilayer. We also found that intercalation of Li or O atoms and carrier injection are effective strategies to manipulate the interlayer magnetic coupling. A magnetic phase transition from AFM to FM can be triggered by atomic intercalations. Interestingly, the Li-intercalated CrI_3_ bilayer showed a more stable FM order than O-intercalated, despite the stronger localization of O 2p orbitals than Li 2s orbital. This abnormal behavior was attributed to a superexchange mechanism. The Curie temperature T_c_ was also higher than the unintercalated. Furthermore, it was found that the interlayer FM coupling and the magnetic anisotropy energy (MAE) could be effectively tuned by charge doping.

## 2. Computational Methods

Density functional theory (DFT) calculations were carried out using the Vienna ab initio simulation package (VASP5.4) [39,40] code with the projector augmented-wave (PAW) method [41]. Exchange–correlation interactions are described by the generalized-gradient approximation (GGA) augmented by Hubbard-U corrections (GGA+U method) in the formalism of the Perdew–Burke–Ernzerhof (PBE) functional [42,43,44]. The calculated on-site Coulomb repulsion and the Hund interaction for Cr 3*d* electrons in the CrI_3_ bilayer were set as U = 2.9 eV and J = 0.7 eV, respectively [37]. An energy cutoff of 450 eV was used for the planewave basis. The convergence threshold and residual force during the self-consistent solution of the Kohn–Sham equations were set as 10^−5^ eV and 0.02 eV/Å, respectively. For the self-consistent calculations, 9 × 9 × 1 and 5 × 5 × 1 Monkhorst–Pack *k*-point grids were adopted for the CrI_3_ bilayer unitcell and a 2 × 2 × 1 supercell. A vacuum layer larger than 15 Å was adopted to eliminate the interaction effects between periodic images. The Monte Carlo (MC) simulation with the Wolff algorithm based on the classical Heisenberg model is used to describe the thermal dynamics of magnetism in equilibrium states [45,46]. Specific heat capacity is calculated by the dissipation-fluctuation theorem. The real-space renormalization group with the majority rule is used to analyze the phase transition and locate the Curie temperature. All of the Monte Carlo algorithms described here were implemented in open source code MCSOLVER [47]. Spin-orbital coupling (SOC) was considered in the calculations of MAE.

## 3. Results and Discussion

**Figure 1:**
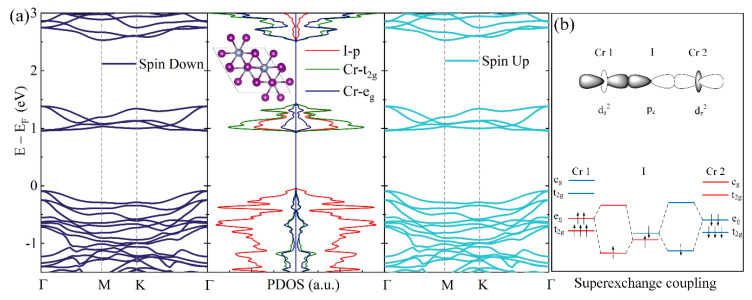
(**a**) Band structures and projected density of states (PDOS) of pristine high-temperature (HT)-phase of the CrI_3_ bilayer, respectively. (**b**) Schematic diagram of the antiferromagnetic (AFM) superexchange coupling mechanism.

The crystal structure of the CrI_3_ bilayer is given in Figure 1a. The optimized lattice constants are *a* = *b* = 6.952 Å, *α* = *β* = 90°, *γ* = 120°. Each Cr atom is coordinated to six I atoms, forming a [CrI_6_] octahedron. As a result of crystal field splitting, Cr 3*d* orbitals split into two subsets, $t_{2g}=\left\{d_{xy},d_{xz},d_{yz}\right\}$ and $e_{g}=\left\{d_{x^{2}-y^{2}},d_{z^{2}}\right\}$. In Figure 1a,b, the electronic structures of the CrI_3_ bilayer are calculated. It was found that the CrI_3_ bilayer showed intralayer FM and interlayer AFM coupling, which is consistent with previous works [48].

In Figure 1b, we proposed a superexchange interaction mechanism to interpret the origin of interlayer AFM coupling in the CrI_3_ bilayer. To investigate the microscopic magnetic coupling mechanism of the CrI_3_ bilayer, the intralayer magnetic coupling (FM coupling) and the interlayer magnetic coupling (*e*_g_–*e*_g_ AFM or *t_2g_–e_g_* FM coupling) were studied, respectively. Monolayer CrI_3_ FM order is due to the near-90° Cr–I–Cr superexchange [15]. Since the distance of the two nearest Cr atoms in the same layer was only about 4 Å, it led to the FM instead of AFM order in the monolayer CrI_3_. On the other hand, the interlayer separation distance was around 7 Å, almost double the intralayer Cr–Cr separation distance. We propose that the interlayer AFM coupling is mainly attributed to a superexchange interaction. In the case of a Cr–I–Cr angle larger than 145 degree, superexchange interaction occurs through virtual electrons hopping between the Cr 3*d* orbitals mediated by the *p* orbitals of two I atoms. Thus, it is expected that both the positions of ions and the layer configuration play crucial roles in determining the resultant interlayer magnetic coupling.

Furthermore, virtual electron hopping is a spin-conserved process. As such, any virtual hopping of Cr 3*d* electrons with the same spin is forbidden in the ferromagnetic state due to the Pauli exclusion principle. Therefore, superexchange pathways that involve the hybridization of two Cr *e*_g_ orbitals must give rise to interlayer AFM exchange (see Figure 1b). The hopping between the interlayer nearest neighbor Cr atoms is realized through *e_g_–I_p_–t_2g_* and the next-nearest-neighbor is realized through *e_g_–I_p_–e_g_* [36]. Analyzing the CrI_3_ bilayer superexchange pathways of the nearest and next-nearest neighbor interlayer Cr atom pairs in the CrI_3_ bilayer, the nearest-neighbor interlayer coupling involves *t*_2g_ and *e*_g_ orbitals, while next-nearest-neighbor coupling involves only *e*_g_ orbitals. Therefore, the resulting ground state in the CrI_3_ bilayer is layered anti-ferromagnetism. The electronic band structures and projected density of states (PDOS) of the CrI_3_ bilayer are given in Figure 1a. The band structure of majority spin is the same as that of minority spin, and the band gap is 1.05 eV. This means that the CrI_3_ bilayer with high-temperature (HT) phase is not magnetic. It is also apparent that the top of the valence band is formed mostly by spin unpolarized p orbitals of the I atoms and the conduction band is formed by the spin unpolarized *t*_2g_ orbitals of Cr.

**Figure 2:**
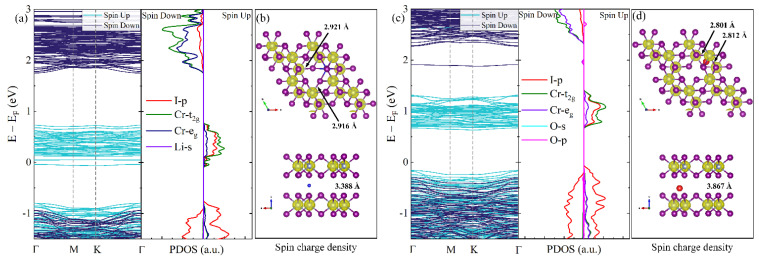
Band structures and PDOS of (**a**) Li and (**c**) O intercalated of 2 × 2 supercell. The (**b**) and (**d**) is the spin charge density and crystal structure parameters. The isosurface value is 0.03 e/Bohr^3^.

In order to manipulate interlayer magnetic coupling of the HT-phase CrI_3_ from AFM to FM, the intercalation of Li and O atoms was applied, as displayed in Figure 2. In Figure 2b, it can be seen that Li is located at directly below I of the upper layer and in the middle between the CrI_3_ bilayer. Unlike the Li intercalation, O deviated below I of the upper layer and was located closer to the underlying CrI_3_, as shown in Figure 2d.

The band structures and PDOS near the Fermi level of Li and O intercalated structures are drawn in Figure 2. It was found that both Li and O intercalation in the CrI_3_ bilayer exhibited spin-polarized states, and the conduction band minimum (CBM) and the valence band maximum (VBM) were both donated from the spin-up state. For the Li-intercalated CrI_3_, the Fermi level was pushed up and across the spin-polarized conduction bands. As a result, the spin-up channel was metallic, while spin-down sub-band remained a sizable band gap of 2.69 eV, which led to the half-metallic character. Unlike the Li intercalated structure, the O intercalated CrI_3_ bilayer maintained a semiconductor, while the band gap was reduced to 0.79 eV. The PDOS of both Li and O intercalated CrI_3_ illustrates that the VBM is mostly contributed by p orbitals of I and the CBM is mostly contributed by d orbitals of Cr. According to the spin charge density in Figure 2b,d, the magnetic moment is provided by Cr atoms. The average magnetic moments of Cr in the Li and O intercalated CrI_3_ bilayer were 3.43 and 3.38 µB, respectively. In addition, the maximum magnetic moments were 3.58 and 3.45 µB, respectively. In short, these results revealed that intercalation had a remarkable influence on the electronic and magnetic characteristics of the CrI_3_ bilayer.

To explore the mechanism for this spin-polarization transition, we compared the crystal structure parameters (lattice constant, interlayer distance, bond length, and bond angle) among the Li intercalated, O intercalated, and pristine CrI_3_ bilayer. As shown in Figure 2b,d, the interlayer distance of Li intercalated decreased from 3.575 Å to 3.388 Å. The Cr–I bond length (2.921 and 2.916 Å) was obviously longer than that of the pristine one (2.764 Å) accordingly. The interlayer distance of O-intercalation was enlarged to 3.867 Å, and the Cr–I bond length was slightly elongated to 2.812 and 2.801 Å. It is expected that these changes in local structures will influence the electronic structure.

In order to explore the preferred magnetic interaction, we defined the exchange energy per formula (f.u.) as

(1) $$\Delta E=(E_{\mathrm{AFM}}-E_{\mathrm{FM}})/n$$

where $E_{\mathrm{AFM}}$ and $\;E_{\mathrm{FM}}$ are the energies of the CrI_3_ bilayer with AFM and FM spin configurations, respectively; and n is the number of CrI_3_ formulas in the supercell. A positive (negative) value of ∆*E* represents FM (AFM) coupling between the two CrI_3_ layers. The interlayer exchange coupling coefficient $J=\Delta E/2{\left|\vec{S}\right|}^{2}$. The oxidation state of Cr in these compounds is expected to be +3. Therefore, from Hund’s rules, we expect that Cr^3+^ has *S* = 3/2. The calculated ∆*E* of the pristine HT-phase CrI_3_ bilayer was as small as −0.17 meV/f.u., which means a weak AFM coupling in the CrI_3_ bilayer and agrees well with the previous work [22]. After the intercalation of Li and O, ∆*E* was calculated as 10.99 and 0.81 meV/f.u., respectively. These results indicate that the magnetic coupling of CrI_3_ bilayer is transformed from AFM to FM. Moreover, ∆*E* of the Li-intercalated CrI_3_ bilayer is much larger than of the O-intercalated or the pristine CrI_3_ bilayer, which means a stronger and more stable FM coupling in the Li-intercalated CrI_3_ bilayer.

Furthermore, based on the Bader charge analysis, the charge distribution in both the Li-intercalation and O-intercalation systems was remarkably different to the pristine one, as listed in Table 1. Obviously, Li transfers 0.867 e to the surrounding I atoms in Li-intercalation, and the O atom obtains 0.871 e from the surrounding I atom in O-intercalation, which brings about an electron/hole doping effect. These results indicate that charge transfer in both Li-intercalation and O-intercalation can significantly affect their original electronic properties.

**Figure 3:**
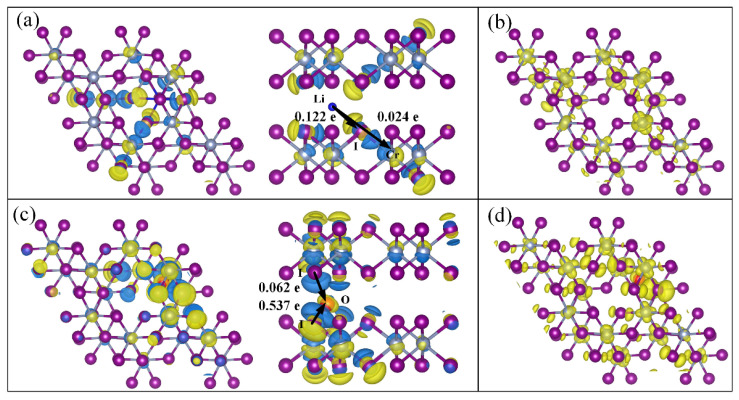
Differential charge density of (**a**) Li and (**c**) O intercalated 2 × 2 supercell. The isosurface value was 0.01 e/Bohr^3^, and the yellow (blue) isosurface contours indicate the charge accumulation (depletion). Band-decomposed partial charge density of (**b**) Li and (**d**) O intercalated 2 × 2 supercell, the isosurface value was 0.001 e/Bohr^3^.

To study the microscopic magnetic coupling mechanism of Li/O intercalated CrI_3_ bilayer. We calculated the differential charge density (∆*ρ*) of both the Li-intercalation and O-intercalation, as shown in Figure 3a,c, respectively, where ∆*ρ* is obtained as follows:

(2) $$\Delta \rho =\rho_{{\mathrm{Li}/O\text{-}\mathrm{CrI}}_{3}}-\rho_{{\mathrm{CrI}}_{3}}-\rho_{\mathrm{Li}/O},$$

where $\rho_{{\mathrm{Li}/O\text{-}\mathrm{CrI}}_{3}}$, $\rho_{{\mathrm{CrI}}_{3}}$, and $\rho_{\mathrm{Li}/O}$ represent the charge density distributions of the Li/O-intercalated, non-intercalated CrI_3_ bilayer, and an isolated Li/O atom, respectively. From Figure 3a,c, it can be seen that the Li atom donates electrons in its intercalated configuration, while the O atom obtains electrons from the nearest I atom. The results are consistent with the Bader charge analysis. For Li-intercalation, the charge density transfer is localized on the interlayer next-nearest-neighbor Cr atoms, which enhances the interlayer *t_2g_–e_g_* FM coupling. For O-intercalation, the charge density transfer is localized near the O atom, which enhances the intralayer FM coupling.

Figure 3b,d illustrate the band-decomposed partial charge densities of Li/O-intercalation, where the isosurface indicates a distribution of electrons at the VBM and a hybridization between the intercalated atom and neighboring atoms. For Li-intercalation, the VBM originates mainly from interlayer Cr–I–Li–I-Cr exchange interactions. The hopping between the interlayer next-nearest-neighbor Cr atoms is realized through *e_g_–I_p_ σ*, *I_p_–I_p_ σ*, and *I_p_–t_2g_ σ* bonding. For O-intercalation, the charge transfers only influenced the intralayer FM coupling, resulting in the hopping between the intralayer Cr atoms through the *e_g_–I_p_–t_2g_* orbitals. Hence, there are two interaction mechanisms that occur in Li/O intercalation: intralayer magnetic coupling and interlayer magnetic coupling. For the Li-intercalation configuration, the interlayer exchange interaction was enhanced due to the enhancement of *t_2g_–e_g_* FM coupling between the next-nearest-neighbor Cr atoms. As a result, the CrI_3_ bilayer switched from semiconducting to half-metallicity by Li intercalation. For the O-intercalation configuration, although the intralayer magnetic coupling was enhanced, the interlayer FM coupling was weaker than that of the Li-intercalation configuration.

**Figure 4:**
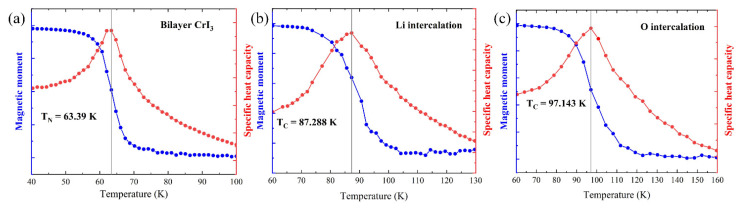
Magnetic moment and specific and specific heat capacity versus temperature in (**a**) pristine CrI_3_ bilayer, (**b**) Li intercalation, and (**c**) O intercalation by Monte Carlo simulation.

To probe the Curie temperature of the CrI_3_ bilayer and the intercalated configuration, we performed MC simulations using a 16 × 16 × 1 matrix, which is based on the classical spin Hamiltonian:

(3) $$H=J_{\mathrm{intra}}\sum_{ij}{\vec{S}}_{i}\cdot {\vec{S}}_{j}+J_{\mathrm{inter}}\sum_{ij}{\vec{S}}_{i}\cdot {\vec{S}}_{j},$$

where $J_{\mathrm{intra}}$, $J_{\mathrm{inter}}$ are the intralayer and interlayer exchange interactions between Cr atoms, respectively. Using the DFT-derived exchange interaction, the Curie temperature T_C_ was predicted by the MCSOLVER code. For each temperature point, the MC simulations involved 8 × 10^4^ sweeps to sufficiently thermalize the system into equilibrium, and the next 6.4 × 10^5^ sweeps per site to acquire the statistical results. The curve of T_C_ is shown in Figure 4. The Neel temperature (T_N_) of the pristine HT phase of the CrI_3_ bilayer was about 63.39 K, which was close to 61 K of the bulk CrI_3_ [5]. After intercalated Li or O atoms, the T_c_ reached up to 87.288 K or 97.143 K. It also proved that the intercalation of atoms can further enhance the spin-polarization of the CrI_3_ bilayer and further increase the Curie temperature.

**Figure 5:**
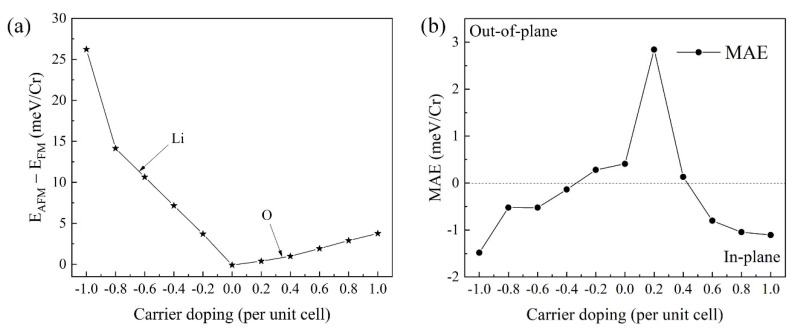
(**a**) Energy difference in the AFM and FM states (*E*_AFM_ − *E*_FM_) for the CrI_3_ bilayer under the carrier doping. Along the *x*-axis, a positive (negative) value represents hole (electron) doping. On the *y*-axis, positive (negative) value of energy represents FM (AFM) coupling in the CrI_3_ bilayer. (**b**) The MAE of the CrI_3_ bilayer with different carrier doping.

In addition, Figure 5a exhibits the energy difference of AFM and FM (*E*_AFM_ − *E*_FM_) for the CrI_3_ bilayer as a function of the carrier doping. It illustrates that the magnetic configuration switched from the interlayer AFM (*E*_AFM_ − *E*_FM_ < 0) to FM (*E*_AFM_ − *E*_FM_> 0) configuration when slightly increasing the carrier doping. Compared with the hole doping, the electron doping significantly changed the energy difference (*E*_AFM_ − *E*_FM_). These results agreed well with that of the Li-intercalation and O-intercalation configurations. The MAE of the CrI_3_ bilayer with different carrier doping is shown in Figure 5b. $\mathrm{MAE}=(E_{x}-E_{z})/n$, where $E_{x}$ and $E_{z}$ are the energies of CrI_3_ bilayer in in-plane [100] and out-of-plane [001] magnetization direction, and *n* is the number of Cr atoms. The positive and negative values of MAE denote that the easy magnetization axis is perpendicular and parallel to the plane of the CrI_3_ bilayer, respectively. Clearly, the MAE can be effectively tuned by the charge doping. The system has a magnetic phase transition from the out-of-plane to the in-plane as *y*-axis after exceeding certain electron doping (∼0.2 per unit cell) and hole doping (~0.4 per unit cell). It should be noted that there was almost no difference in energy with respect to the different in-plane directions from our DFT calculations.

## 4. Conclusions

In summary, we demonstrated computationally that the interlayer magnetic coupling of the CrI_3_ bilayer could be well controlled by intercalation and carrier doping. Interlayer atomic intercalation and carrier doping were proven to induce an antiferromagnetic (AFM) to ferromagnetic (FM) phase transition for the spin-polarization in the CrI_3_ bilayer. Our results revealed that the AFM to FM transition induced by atom intercalation is a result of enhanced superexchange interaction between Cr atoms of neighboring layers. FM coupling induced by O intercalation mainly originated from the improved superexchange interaction mediated by Cr 3d–O 2p coupling. FM coupling induced by Li intercalation was found to be much stronger than that by O intercalation, which was attributed to the much stronger superexchange by electron doping than by hole doping. The stronger interlayer ferromagnetic coupling generally means higher Curie temperature. This comprehensive spin exchange mechanism was further confirmed by our results of the carrier doping effect on the interlayer magnetic coupling. Our work provides a deep understanding of the underlying spin exchange mechanism in 2D magnetic materials.

## Figures and Tables

**Table 1:** Charge transfer based on the Bader charge calculation in the pristine high-temperature (HT) phase of the CrI_3_ bilayer, Li intercalated, and O intercalated HT phase in 2 × 2 supercell, respectively. A negative value indicates charge accumulation on the respective atom, while a positive value indicates charge depletion.

	Pristine CrI_3_ Bilayer		Li Intercalation			O Intercalation		
Atom	Cr	I	Cr	I	Li	Cr	I	O
Charge transfer	1.067	−0.356	1.043	−0.478	0.867	1.035	0.537	−0.871

## Data Availability

The data presented in this study are available upon reasonable request from the corresponding author.
